# Cannabinoids from inflorescences fractions of *Trema orientalis* (L.) Blume (Cannabaceae) against human pathogenic bacteria

**DOI:** 10.7717/peerj.11446

**Published:** 2021-05-13

**Authors:** Tiwtawat Napiroon, Keerati Tanruean, Pisit Poolprasert, Markus Bacher, Henrik Balslev, Manop Poopath, Wichai Santimaleeworagun

**Affiliations:** 1 Department of Biotechnology, Faculty of Science and Technology, Thammasat University, Pathum Thani, Thailand; 2 Biology program, Faculty of Science and Technology, Pibulsongkram Rajabhat University, Phitsanulok, Thailand; 3 Institute of Chemistry of Renewable Resources, University of Natural Resources and Life Sciences Vienna (BOKU), Tulln an der Donau, Austria; 4 Ecoinformatics Section, Department of Bioscience, Faculty of Science and Technology, Aarhus University, Aarhus, Denmark; 5 Department of National Parks Wildlife and Plant Conservation, The Forest Herbarium, Forest Botany Division, Bangkok, Thailand; 6 Faculty of Pharmacy, Silpakorn University, Department of Pharmacy, Nakhon Pathom, Thailand

**Keywords:** Infectious diseases, Cannabinoids, Botany, Chromatography, Cannabaceae

## Abstract

**Background:**

Cannabinoids; tetrahydrocannabinol (THC), cannabidiol (CBD) and cannabinol (CBN), might show antibacterial activity. *Trema orientalis* is a species in the Cannabaceae that is closely related to *Cannabis* through plastome phylogenetic evidence. This species is widely distributed throughout tropical Asia and is used as traditional medicine, particularly for the treatment of infectious diseases. However, no studies on the antibacterial activity of cannabinoid-containing inflorescences extracts are available. Thus, the aim of this study was to determine cannabinoid content and antibacterial activity of inflorescences fractions from *T. orientalis* native to Thailand.

**Methods:**

We hypothesized that inflorescences from *T. orientalis* might display cannabinoids similar to *Cannabis* because of their close taxonomic relationship. We extracted the mature inflorescences and infructescence of *T. orientalis* in three disparate populations from different Thailand floristic regions. Extractions were subsequently partitioned into hydrophilic and lipophilic fractions using distilled water and chloroform. The lipophilic extracts were further fractionated by the column chromatography with gradient elution and analyzed by gas chromatography-mass spectrometry (GC-MS). Characterized cannabinoids were used in bioassays with multidrug-resistance bacteria.

**Results:**

Lipophilic extracts and fractions of inflorescences from all Thailand floristic regions consistently displayed cannabinoids (THC, CBD and CBN) in various quantities. These extracts exhibited inhibitory activity for *Staphylococcus aureus*, *Pseudomonas aeruginosa*, and *Acinetobacter baumannii* strains with minimum inhibitory concentration values varying from 31.25 to 125 µg/mL.

**Conclusion:**

Our study is the first to report cannabinoid detection in extracts from inflorescences of *T. orientalis,* a species in the Cannabaceae. These extracts and their fractions containing cannabinoids showed pronounced antibacterial activity. The use of analytic methods also demonstrated reproducible cannabinoid extraction.

## Introduction

Major cannabinoids, including tetrahydrocannabinol (THC), cannabidiol (CBD) and cannabinol (CBN), are chemotaxonomic markers in inflorescences of the genus *Cannabis* (Cannabaceae). These natural products have been used for centuries to treat disease and alleviate pain (McPartland, 2018). Use of plant species in Cannabaceae as sources for antibacterial and antiviral agents in pulmonary or respiratory diseases has been reported (Caiaffa et al., 1994; Roth et al., 2004; Appendino et al., 2008). Naturally, cannabinoids in *Cannabis* might be from two ways including production by its own in plant or stimulated plant by environmental factors to initiate chemical isomerization. Typically, cannabinoids are analyzed by capillary gas chromatography with mass spectrometry (GC-MS) (Yotoriyama et al., 2005). Cannabinoid treatment for bacteria infection was reported from clinical trials using a combination of cannabidiol (CBD) and the antibiotic bacitracin (BAC). This combination effectively treated infections caused by Gram-positive resistant bacteria (Wassmann, Højrup & Klitgaard, 2020).

*Trema orientalis* is a species closely related to a sister group of *Cannabis* (Jin et al., 2018). The species is a common natural pioneer used in traditional medicine for the treatment of infections in tropical regions, particularly in continental Asia (Adinortey, Galyuon & Asamoah, 2013). This species is extensively used to reduce fevers and fight infection. Phytochemically, *T. orientalis* displays different major constituents in different plant parts—tremetol, simiarenol and simiarenone in leaves; tremetol, swertianin, scopoletin and several fatty acids and glycosides in stem bark; sterols and fatty acids in roots (Adinortey, Galyuon & Asamoah, 2013; Parvez et al., 2019). However, compounds from inflorescences have not been assessed in previous reports.

*Trema orientalis* is used for treatment of many ailments involving antibacterial and antiviral infection in the respiratory system (Watt & Breyer-Brandwijk, 1962). Treatment of infections caused by multidrug-resistance bacteria (MDR), including *Staphylococcus aureus*, *Pseudomonas aeruginosa* and *Acinetobacter baumannii,* is challenging, and newly emerged and extremely drug-resistant strains have posed serious problems in public health care (WHO, 2017). Antimicrobial agents from medicinal plants modify activity exhibited by MDR strains, yet medicinal plants are still underused as sources of antimicrobial agents. Antibacterial activity of inflorescence extracts from *T. orientalis* species is based on traditional uses in respiratory infection. This study thus investigates the content of cannabinoids as chemical markers of the *Cannabis* that might also be found in inflorescence extracts of *T. orientalis*. Also, we detail the activity of these extracts toward MDR bacteria that cause respiratory infection. We hypothesized that inflorescence extracts from *T. orientalis* might contain cannabinoids and might display antibacterial effects. We analyzed extracts and tested their antibacterial activity. Our study is the first to report cannabinoids from the inflorescence extracts of *T. orientalis* in the Cannabaceae.

## Materials & Methods

### Plant materials

Nine specimens of *Trema orientalis* were collected from three floristic regions in northern, southeastern and peninsular or southern Thailand. Locations were selected from distribution notes for herbarium specimens. All samples were identified by using comparative macro and micro-morphology using key characteristics in the Flora of Thailand and related documents. Voucher specimens were deposited in the BKF herbarium and indexed Index Herbariorum (Thiers, 2020). We selectively collected mature inflorescences flowers from November, 2019 to February, 2020 during the flowering period in agricultural and disturbed areas.

### Extraction procedures

Mature inflorescences of *T. orientalis* were air-dried without sunlight. Dried materials (200 g) were powdered using a blender before phytochemical analysis. Powders were macerated in 500 mL of methanol for 10 days in the dark at room temperature. Extracts were filtered and concentrated under a vacuum in a heating bath at 45 °C and 218 mbar to acquire crude extracts. Extract was partitioned in 250 mL of distilled water (hydrophilic fraction) and chloroform (lipophilic fraction) in a separatory funnel flask of 500 mL. Finally, sediment was allowed to settle for 30 min in a fume, and the chloroform fraction was selectively collected and evaporated to dryness for further experiments.

### Compound fractionation

The lipophilic extract contained 680 mg dry weight of inflorescences extract; 200 g was separated using column chromatography (glass column size: 1.7 × 80 cm) with 60 g of silica gel (0.04–0.063 mm, Merck) as the absorbent. The gradient solvent system was mixed 100 mL per fraction in three steps with increasing polarity: (1) hexane and diethyl ether mixed in 5 fractions (95:5, 90:10, 75:25, 50:50 and 0:100%v/v), (2) diethyl ether and methanol also mixed in 5 fractions (100:0, 75:25, 50:50, 25:75 and 10:90%v/v) and (3) One fraction of 100% methanol. All fractions from each gradient were collected in 25 mL sub-fractions then separated on TLC plates with hexane:ethyl acetate (8:2 v/v). These TLC plates were visualized under UV light (wavelengths 254 and 365 nm) and anisaldehyde sulfuric acid spray for terpenoid detection. HPLC chromatographic analysis for observed compounds completed and used to combine fractions into ten sub-fractions with similar characteristics. Sub-fractions were evaporated, collected in diethyl ether and stored at −20 C. Dry residues showed different physical characteristics.

### Chromatography procedures (HPLC and GC-MS)

Subsequently, high-performance liquid chromatography (HPLC) analysis in each sub-fraction used a PerkinElmer (Flexar series) with a UV photodiode array detector at wavelengths of 230, 254 and 280 nm. A reverse-phase BDS hypersil™ C18 (thermo scientific™) column, (250 × 4.6 mm) was used for separation. The solvent system consisted of an aqueous buffer (A) containing (0.015 M ortho-phosphoric acid pH 3 and 0.0015 M tetrabutyl ammonium hydroxide) and methanol (B) (HPLC analytic grade, Merck). Sub-fractions were prepared at a concentration of 10 mg/mL in methal (analytic grade, Merck); 10 µL of solutions was injected for all analyses. Solvent flow rate was 1 ml min^−^^1^ within 30 min for chromatogram recording. The mobile phase started at 60% B for 16 min and then increased to 90%–100% B within 6 min, with 100% B continuing for 6 min.

Gas chromatography-mass spectrometry (GC-MS) used an Agilent Technologies Model 6890N coupled with a 5973 inert mass spectrometer (Agilent Technologies, USA). Compounds were separated on an Agilent Technologies™ column DB5MS cross-linked poly 5% diphenyl 95% dimethylpolysiloxane (0.25 mm ×30 m ×0.25 µm film thickness). Column temperature was initially 45 °C was then increased to 100 °C for 1 min and was raised to 300 °C at a rate of 10 °C/min and held for 9 min. Helium was used as the carrier gas at a flow rate of one mL/min. The injector, transfer line and ion-source temperatures were 250, 280, and 230 °C, respectively. MS detection was performed with electron ionization at 70 eV, operating in the full-scan acquisition mode in the range of 30–450 m/z and 150 °C MS quadrupole temperature (Ruppel & Kuffel, 2017). GC-MS results provided Total Ion Chromatogram (TIC) information including retention time, cannabinoid contents, limited of detection (LOD), percentage of maximum corrected area (% max.corr.) and percentage of total area (% total areas).

### Analytical standards

Standard 1,000 µg/mL solutions for cannabinoids (THC, CBD and CBN, Merck™) were used to compare with sample results. GC-MS analytical processes in report no. TRCM63 was certified by Central Laboratory (Thailand) Co., Ltd. using the standard of ISO/IEC 17025. Each sub-fraction (990 µL) was mixed with 10 µL of three cannabinoid internal standards for calibration (modified from Ruppel & Kuffel, 2017). The results were reported as percent w/w of cannabinoids. The reproducibility was based on retention time and peak areas of standards, and sensitivity was determined by the LOD.

### Antibacterial activity

Four MDR bacteria (superbugs) in the WHO list, *Staphylococcus aureus* ATCC 43300 (methicillin-resistant), *Staphylococcus aureus* ATCC 25923, *Pseudomonas aeruginosa* ATCC 27853 and *Acinetobacter baumannii* ATCC 19606, were obtained from the Department of Medical Science, Ministry of Public Health, Thailand.

Disk diffusion assays were used for antibacterial testing of lipophilic sub-fractions. The MDR bacterial strains were inoculated into Muller-Hinton broth (MHB) (Oxoid™, Hamshire, UK). Bacterial cell suspensions equivalent in density to 0.5 McFarland (1.5 × 10^8^ CFU/mL) were then inoculated with Muller-Hinton agar (MHA-Difco™, USA). For the compound loading, sub-fraction (200 µg) were purged with nitrogen dioxide (NO_2_), then dissolved with 20% dimethyl sulfoxide (DMSO; Sigma-Aldrich, St Louis, USA) in distilled water (DW) at an amount of 100 µL. The paper disks (six mm diameter) were soaked in the sub-fraction and placed 30 mm apart on MHA containing the test bacteria. The MHA plate was then incubated at 37 °C for 18 h.

A broth microdilution assay was used to measure minimum inhibitory concentration (MIC) of various sub-fractions using serial dilutions in dimethyl sulfoxide (DMSO; Hybri-Max™, Sigma-Aldrich, USA), ranging from 1024 to 0.5 µg/mL, in 96-well microplates with the subsequent addition of a standard inoculum of test bacteria in Muller-Hinton™ broth (MHB). All samples were duplicated and incubated at 37 °C for 18 h. MIC was determined as the lowest concentration of extract that reduced the turbidity of cultures. The quality control followed the Clinical and Laboratory Standards Institute (CLSI, 2019) guidelines for standardized sensitivity tests. Bacteria were tested against several antibiotics as positive controls: PIP/TAZ (Piperacillin/Tazobactam 100/10 µg), AMK (Amikacin 30 µg), Ceftriaxone (30 µg), CTX (Cefotaxime 30 µg), Ceftazidime (30 ×), CIP (Ciprofloxacin 5 µg), Imipenem (10 µg), TMP/SMX (Trimethoprim/Sulfamethoxazole 1.25/23.75 µg) and VAN (Vancomycin 30 µg). All bacterial strains were cultivated at 37 °C for 18 h. The resulting inhibition zones (diameter of clear zone; mm) were compared to those of standard antibiotics in the (CLSI, 2019) guideline (Table S3).

## Results

### Determination of cannabinoids in *Trema orientalis* inflorescence extracts

Fractions displaying similar TLC chromatograms were combined into sub-fractions for subsequent testing. Fractions 9 and 10 were combined into S1, fractions 11 and 12 into S2, fractions 13 to 18 into S3 and fractions 19 and 20 into S4. Terpenoids were detected on developed TLC plates using anisaldehyde sulfuric acid reagent. Only sub-fraction S3 showed two dominant violet spots at Rf values of 0.5 and 0.8, respectively.

**Figure 1:**
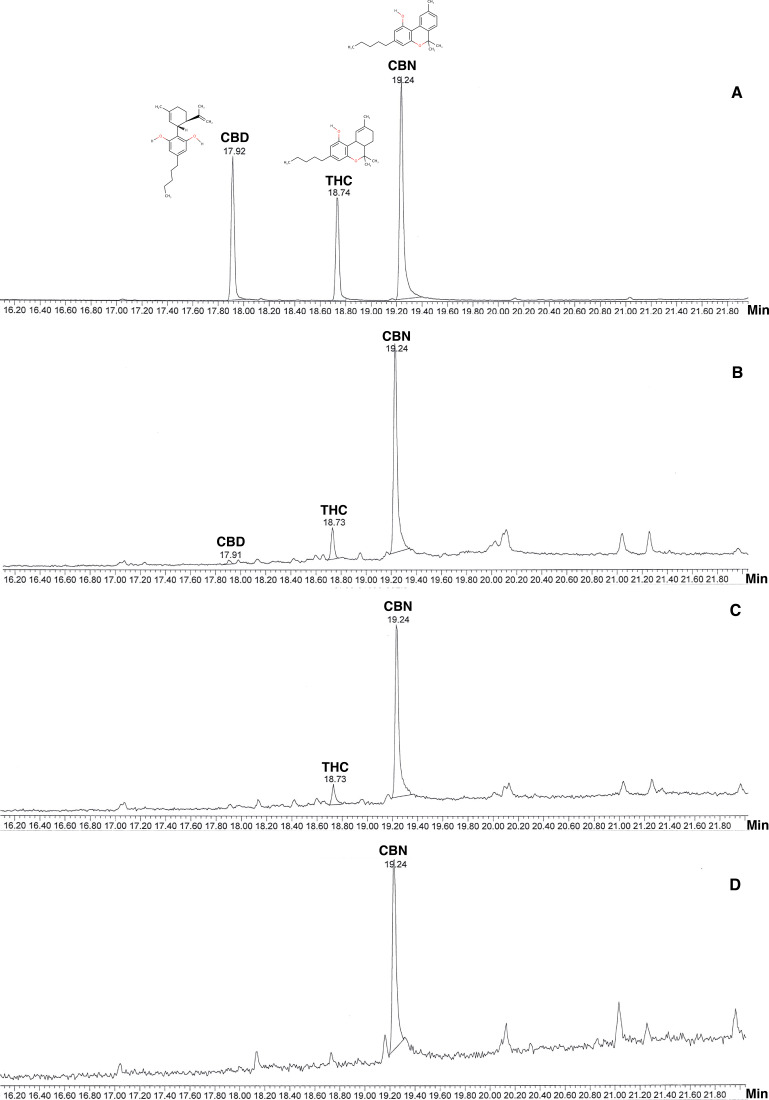
Total Ion Chromatogram (TIC) from GC-MS of cannabinoids compared with internal standard. (A) Internal standard of cannabinoids; (B) Inflorescences fraction from Southern or Peninsular; (C) Inflorescences fraction from Northern; (D) Inflorescences fraction from Southeastern.

TICs from GC-MS showed THC (B), CBD (C) and CBN (D) with precise retention times (RT) (Fig. 1). Cannabinoids identified in *T. orientalis* inflorescence sub-fraction (S3) showed the highest levels of CBN in all samples with lower concentrations of THC and CBD. The highest yields of cannabinoids were found in different regions, including the highest CBN (357.46 mg/kg, RT 19.24 min) and THC (89.96 mg/kg, RT 18.74 min) from the northern region and CBD (5.22 mg/kg, RT 17.92 min) from the southern region; THC and CBD were not present in samples from the southeastern region. However, some samples from all regions displayed some cannabinoids in similar inflorescence extracts (Table 1).

**Table 1:** Cannabinoids were compared with internal standards, and *T. orientalis* inflorescence fractions from plants collected from different regions in Thailand.

Region/Province	Collector no.	Habitats	Cannabinoids detection (mg/kg)^*^		
			THC	CBD	CBN
**Northern (N)**					
Uttaradit	TN et al. 011	Agricultural areas	89.96	–	357.46
Phitsanulok	TN et al. 009	Forest edges	70.17	–	324.05
Phetchabun	TN et al. 012	Disturbed areas	–	–	188.49
**Southeastern (SE)**					
Chanthaburi	TN et al. 008	Beach forest	–	–	51.63
Rayong	TN et al. 015	Beach forest	–	–	55.84
Trat	TN et al. 010	Beach forest	–	–	50.06
**Southern or Peninsula (PEN)**					
Chumphon	TN et al. 007	Agricultural areas	38.13	5.22	132.18
Nakhon Si Thammarat	TN et al. 014	Disturbed areas	–	–	175.69
Songkhla	TN et al. 013	Disturbed areas	30.12	2.00	140.19

**Notes.**

* An asterisk refers to cannabinoids in inflorescences fractions (10 mg/mL concentrations) dissolved with methanol and LOD was established as 0.5 with GC-MS.

### Antibacterial activity of cannabinoids fraction

**Figure 2:**
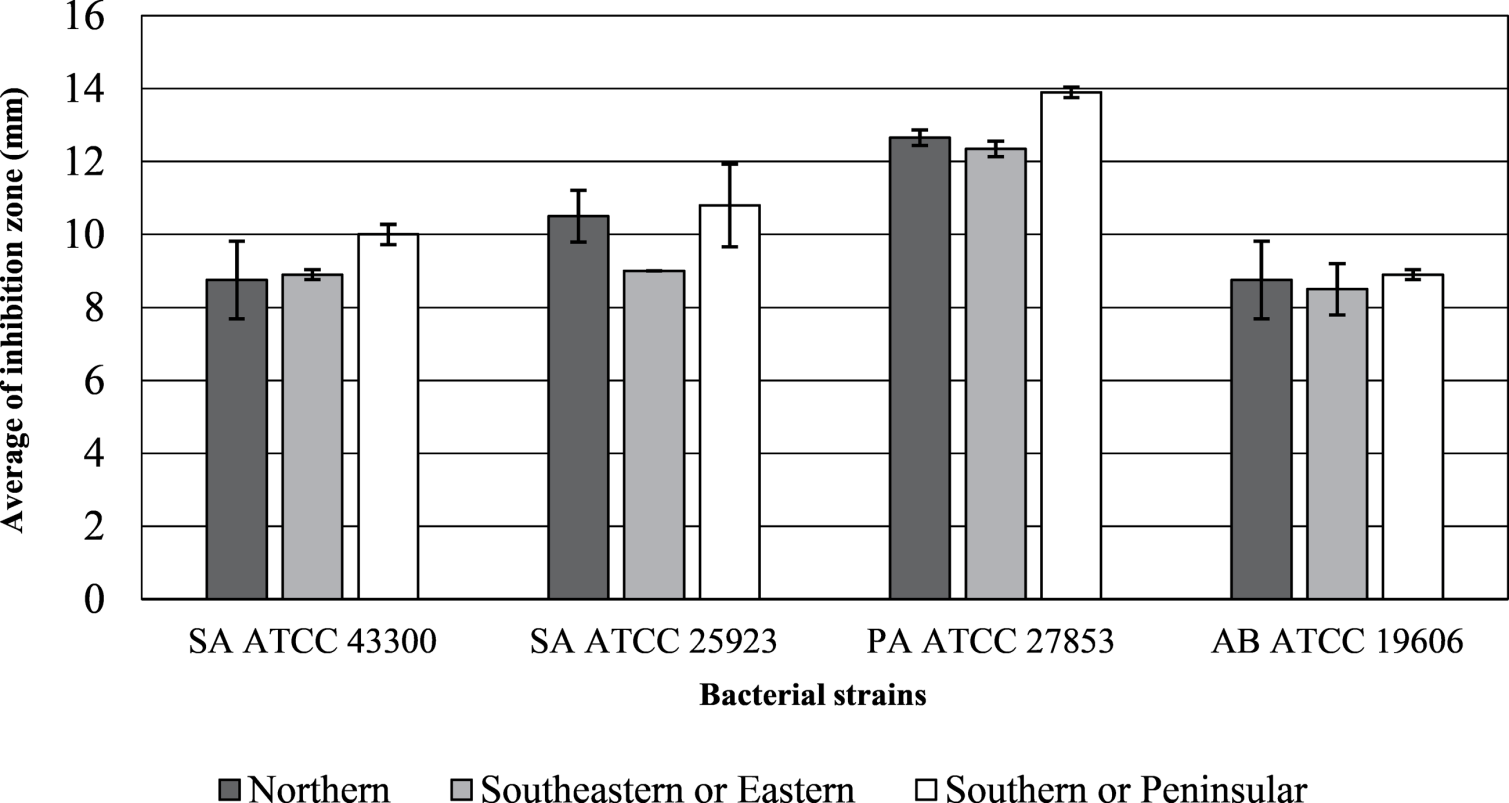
Inhibition zone diameters (mm) of inflorescence fraction (S3). Diameter > six mm indicates inhibition zone.

**Figure 3:**
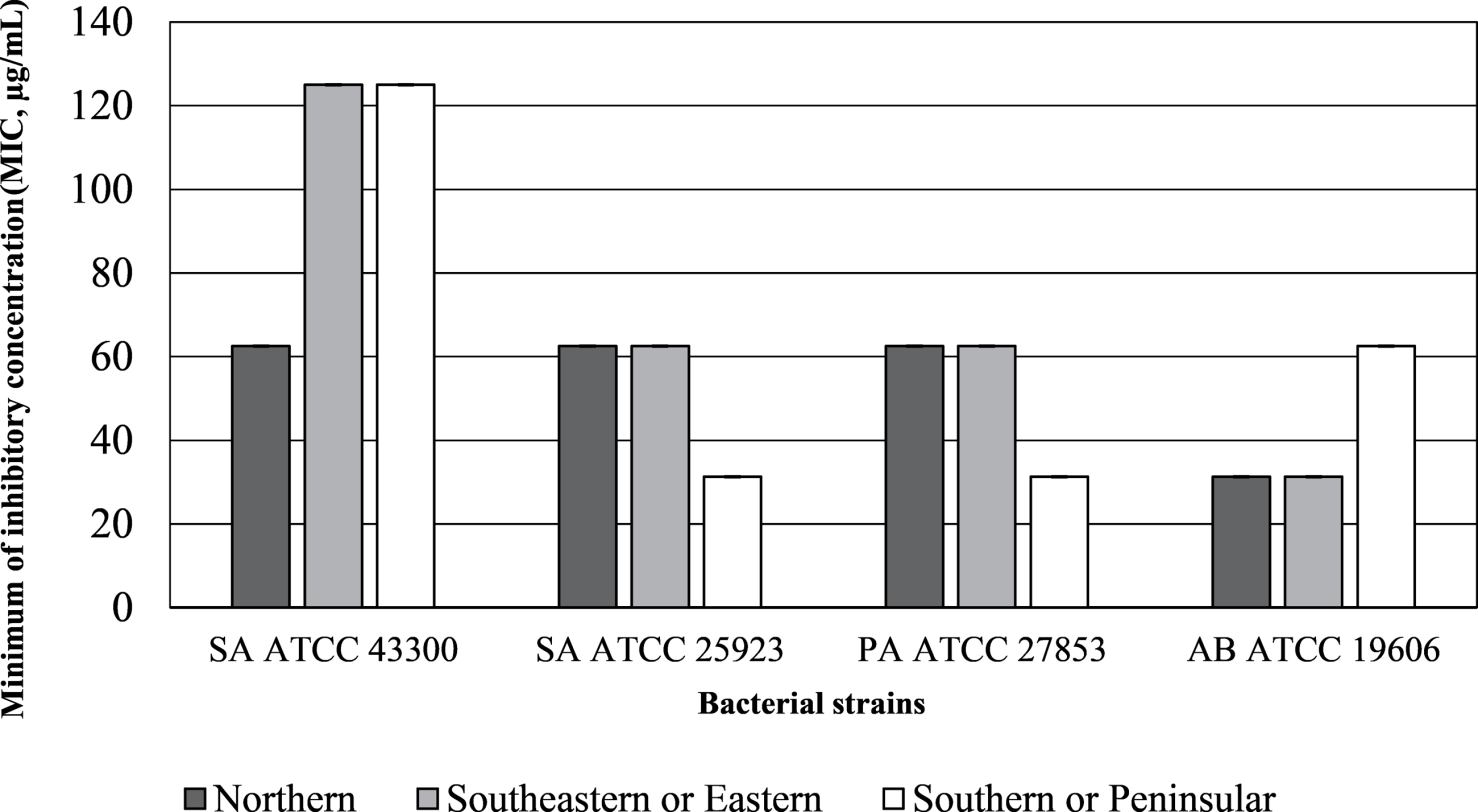
Minimum inhibitory concentration (MIC, µg/mL) of inflorescence fraction (S3).

The inhibition zone diameters and MIC values of the inflorescence sub-fraction S3, which contained cannabinoids, are presented in Figs. 2 and 3. Sub-fraction S3 from plants collected from all regions showed antimicrobial activity against MDR bacteria, with zones of inhibition measuring 8 to 14 mm (Table S1). However, sub-fractions S1, S2 and S4 showed little or no growth inhibition (MIC > 1,024 µg/mL) and no clear zone of inhibition on MHA media. The S3 sample from the northern region displayed the lowest MIC concentration across all bacterial strains. Lowest MICs for S3 indicated significant antibacterial activity against *Staphylococcus aureus* ATCC 43300 (64.25 µg/mL), *S. aureus* ATCC 25923, *P. aeruginosa* ATCC 27853 and *A. baumannii* ATCC 19606 (31.25 µg/mL). MDR strains of *P. aeruginosa* ATCC 27853 (AmpC *β*-lactamase producing strain) and *A. baumannii* ATCC 19606 were inhibited by S3 that contained cannabinoids in samples from each region.

### Trend of occurrence cannabinoids in the studied *Trema orientalis*

**Figure 4:**
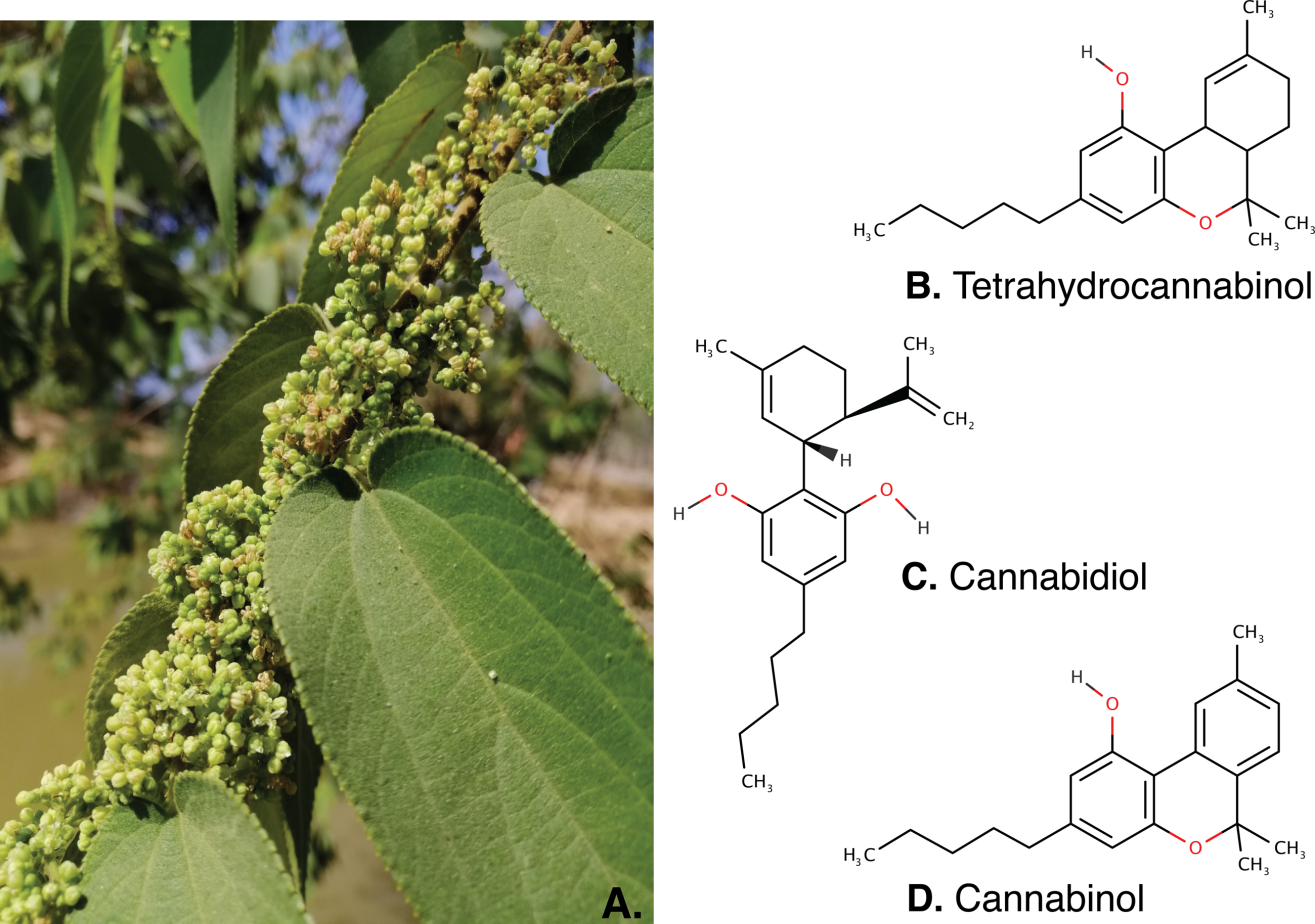
The inflorescence of *Trema orientalis* (A) and major cannabinoids structure; Tetrahydrocannabinol (B), Cannabidiol (C) and Cannabinol (D) were found in inflorescence fractions.

The occurrences of cannabinoids; THC, CBD and CBN in the inflorescence of *T. orientalis* collected from different regions are provided in Table 1 and inflorescence characters which found these compounds displayed in Fig. 4. Cannabinoids were qualitatively and quantitatively detected in all samples at varying concentrations in different regions. Hence, the presence of cannabinoids in inflorescence extracts may be a chemical marker for several members in the Cannabaceae. Variation in cannabinoid content across regions may be due to differences in natural biotic or abiotic factors that affect cannabinoid production, such as geographical habitats and unequal seasonal periods.

## Discussion

### Cannabinoids detection and botanical relationship

Our study is the first to report cannabinoids (THC, CBD and CBN) detection and antibacterial activity in extracts from inflorescences of *T. orientalis,* family Cannabaceae. Cannabinoids are widely distributed in the Cannabaceae, particularly in the genus *Cannabis* (*Cannabis sativa* and *Cannabis indica*) which is important in drug development for its anesthetic, antipsychotic properties and antibacterial activity against MDR bacterial strains (Kostic, Pejic & Skundric, 2008; Appendino et al., 2008; Marks et al., 2009). Cannabinoids are in the terpenoids group dominantly distributed in *Cannabis*. These compounds are concentrated on trichomes on flower surfaces of inflorescences (Backer et al., 2012; Wang et al., 2017). Cannabinoids have not been reported in other plant families, and show broad pharmacological properties (Thomas & Elsohly, 2016).

The major cannabinoids are tetrahydrocannabinol (THC) which is the main psychoactive compound, cannabidiol (CBD) as an anticonvulsant and anti-inflammatory drug in development, and cannabinol (CBN) that exhibits sedative and anticonvulsant properties (Paolino et al., 2016; Domingos et al., 2017; Borille et al., 2017). Total cannabinoid content of *Cannabis* inflorescences is 15.77%–20.37% of dry weight using modern drug analysis (Jin et al., 2020). occurrences of major cannabinoids in inflorescences of *T. orientalis* is first reported in this study. *T. orientalis* inflorescences have been used to treat bacterial bronchitis, pneumonia and pleurisy in children (Watt & Breyer-Brandwijk, 1962; Adinortey, Galyuon & Asamoah, 2013). However, inflorescences of *T. orientalis* lack of chemical investigation and GC-MS cannabinoid detection is not previously reported. Presence of these compounds in a species other than *Cannabis* will help interpret plant distribution and botanical relationships.

Most genera in the Cannabaceae have persistent tepals or perianth and stigmas in the mature flower at the end of the flowering season, including *Cannabis*, *Humulus*, *Parasponia* and *Trema* (Yang et al., 2013; Zhang et al., 2018). Also, these genera show characteristic features, such as glandular and non-glandular trichomes. Trichomes are important for the accumulation of several terpenoids, including cannabinoids. These compounds are mostly produced in glandular trichomes, particularly in the genus *Cannabis*. However, trichomes are characteristics of flowers in the family. An update of Cannabaceae by *Zhang et al.* in 2018 based on plastome assembly showed strong bootstrap support (BP = 100) for a monophyletic group. The *Trema* and *Parasponia* clade and the *Humulus* and *Cannabis* clade are sister groups with strong bootstrap support (BS = 100). These close relationships led us to explore cannabinoids in the genus, *Trema*.

Current cannabinoid analytics allow rapid and precise detection. TLC is expected to meet the The Association of Official Agricultural Chemists (AOAC) needs for use in cannabinoid industries in the future (Sherma & Rabel, 2019). TLC was developed in the United States and worldwide, together with consensus methods by organizations including American Oil Chemist’s Society (AOCS), USP (United States Pharmacopeial Convention), and ASTM International (American Society for Testing and Materials) working with scientists and stakeholder communities (Audino, 2018). We used TLC for terpenoid screening with visualization by specific reagent spraying (anisaldehyde sulfuric acid). This approach allows positive testing for the presence of cannabinoids as odorless terpenoids. Preliminary phytochemical screening using TLC is important for detecting terpenoids in extracts. The approach allowed cannabinoid detection at a reduced cost. Our results on TLC screening are consistent with terpenoid and cannabinoid investigation reported by Sherma & Rabel (2019). These authors showed positive results for cannabinoids and terpenoids with TLC and a similar solvent system (hexane:ethyl acetate, 8:2 or 4:1 v/v) in a saturated chamber with specific reagent spraying and characteristic color patterns.

GC-MS was used for cannabinoids using internal standards under ISO/IEC 17025 laboratory certification in Thailand. GC-MS and its combination with other mass spectrometry allow separation and identification of cannabinoids from the National Institute of Standards and Technology Library (Mariotti et al., 2016; Calvi et al., 2018). Our acquired chromatogram peaks of cannabinoids in *T. orientalis* extracts are well defined, and the order of elution is similar to that reported in the *Cannabis* literature (Mariotti et al., 2016; Broséus, Anglada & Esseiva, 2010; Hazekamp et al., 2005). Similar retention time was found among internal cannabinoid standards and extracts in GC-MS analysis with a stationary phase, non-polar column (DB5 column, 5% diphenyl-95% dimethylpolysiloxane) (Santos et al., 2019). These column properties are similar the column used in the present study and internal standards provided different retention times (CBD =17.92 min, THC =18.74 min and CBN =19.24 min) (Fig. 2).

### Antibacterial activities

Liquid from *Trema orientalis* inflorescence crushing has long been known to treat bronchitis, pneumonia and pleurisy symptoms in children (Watt & Breyer-Brandwijk, 1962). These symptoms are related to bacterial infection in the respiratory system. Important bacteria, such as *Staphylococcus aureus*, *Pseudomonas aeruginosa* and *Acinetobacter baumannii,* are often resistant to antimicrobials (WHO, 2017). Development of novel antibacterial agents against MDR strains is difficult due to the presence of decreased membrane permeability and multidrug efflux pumps (Morita, Tomida & Kawamura, 2014). We found cannabinoids in inflorescence extracts of *T. orientalis*, cannabinoids show antibacterial properties, inhibiting the growth of a variety of methicillin-resistant *Staphylococcus aureus* (MRSA) strains, which are clinically relevant strain (Appendino et al., 2008). Similarly, our inflorescence extracts inhibit pathogenic bacteria, particularly MDR strains (Appendino et al., 2008; Adinortey, Galyuon & Asamoah, 2013). Cannabinoids were developed as modulators of lipid affinity for the olivetol core of a poorly active antibacterial pharmacophore, supporting antibiotic development; major cannabinoids show potent activity against MDR bacteria (Appendino et al., 2008). The S3 fraction showed potent antibacterial activity, with the clear zone on disk diffusion assay in the range of 8 to 14 mm that is similar to the ranges of inhibition zones of 8 to 25 mm which cannabinoids contained in mouthwash products (Vasudevan & Stahl, 2020). For MIC values, the lowest concentration of extract in the range of 31.25 to 125 µg/mL, cannabinol (CBN) was found in all samples in our study. These results conform with antibacterial cannbinol (CBN) in Cannabis (Appendino et al., 2008) that shows promise in fighting MRSA bacteria with low MIC concentrations of less than or equal to specifically resistant antibiotics such as Amikacin (30 µg) and Piperacillin/tazobactam (100/10 µg). Our observation suggests that cannabinoids in this study are the most likely antibacterial activity extension found in *T. orientalis* inflorescences preparation. Generally, CBN is less abundant in *Cannabis* than THC and CBD (Izzo et al., 2009), but in our study CBN was the major cannabinoid. CBN is reported in skin treatment for MRSA infection (Appendino et al., 2008), consistent with our results showing substantially high / and showed substantially high CBN content and significant antibacterial properties. Activities were notable for all four strains, particularly in *S. aureus* ATCC 25923, *P. aeruginosa* ATCC 27853 and *A. baumannii* ATCC 19606 which show high resistance for *β*-lactams antibiotics. These findings are similar to a previous report of antibacterial properties of *T. orientalis (Uddin, 2008)*. Efficacy against *P. aeruginosa* and *A. baumannii*, the major epidemic MDR strains occurring in Thailand and Southeast Asia hospitals, is noteworthy (Werarak et al., 2012).

Fractions that contained cannabinoids were mixtures of active compounds; synergistic effects for compounds could be more effective than any single constituent (Rasoanaivo et al., 2011). Synergistic effects of mixed compounds may act as antibacterial agents for resistance strains (Gilbert & Alves, 2003). Thus, fractions that contained cannabinoids of *T. orientalis* can be considered natural antibacterial agents for future antibiotic development for specific respiratory infections that conform with traditional use.

## Conclusions

Our findings are the first to report cannabinoids contained in *Trema orientalis,* which is closely related to *Cannabis*. Inflorescence extracts of *T. orientalis* have the potential to be used as antibacterial agents, particularly for respiratory infection caused by *Staphylococcus aureus*, *Pseudomonas aeruginosa* and *Acinetobactor baummanii*. Analytical methods based on gas chromatography-mass spectrometry (GC-MS) was used for precise and sensitive cannabinoid detection. TLC and HPLC were used for qualitative screening to help with cost management and rapid investigation. Cannabinoid detection found cannabidiol (CBD), tetrahydrocannabinol (THC) and cannabinol (CBN) in inflorescences of *T. orientalis*. The occurrence of cannabinoids in *T. orientalis* shows the relationship between genera within the Cannabaceae. Therefore, continuing study in *T. orientalis* inflorescences is required to detect cannabinoids in other species in the genus *Trema* to identify chemotaxonomic characteristics for grouping within the cannabis family. Our work might also lead to the introduction of *T. orientalis* in agricultural areas as a model for inflorescence sources for supporting antibacterial agents in the future.

##  Supplemental Information

10.7717/peerj.11446/supp-1 Supplemental Information 1 Inhibition zone (mm) of *T. orientalis* inflorescence fractions from plants collected from different regions in Thailand against bacterial strains **Note:** *Each sample was tested in duplicate: S1 = Sample 1 and S2 = Sample 2 **Abbreviation of bacterial strains: SA ATCC 43300 = *Staphylococcus aureus* ATCC 43300 (methicillin-resistant), SA ATCC 25923 = *Staphylococcus aureus* ATCC 25923, PA ATCC 27853 = *Pseudomonas aeruginosa* ATCC 27853 (AmpC *β*-lactamase producing strain) and AB ATCC 19606 = *Acinetobacter baumannii* ATCC 19606.

10.7717/peerj.11446/supp-2 Supplemental Information 2 The inhibition effects of standard antibiotics with control species by the CLSI (2019) **SA ATCC 25923**: *Staphylococcus aureus* ATCC 43300. **SA** **ATCC 43300**: *Staphylococcus aureus* ATCC 43300. **KP** * ** ATCC 700603**: *K. pneumoniae* ATCC 700603 and ** PA** * ** ATCC 27853**: *Pseudomonas aeruginosa* ATCC 27853. These strains were used as control species; the inhibition zone in each antibiotic was within the quality control ranges set by the CLSI (2019)*. NT abbreviation was refer to not tested.
*The quality control ranges set by the CLSI (2019): For *K. pneumoniae* strain ATCC 700603, the different clear zone diameter of antibiotics including ceftazidime (10–18 mm), cefotaxime (17–25 mm), and ceftriaxone (16–24 mm). For *P. aeruginosa* strain ATCC 27853, the different clear zone diameter of antibiotics including piperacillin/tazobactam (25–33 mm), amikacin (18–26 mm), and ciprofloxacin (25–33 mm).

10.7717/peerj.11446/supp-3 Supplemental Information 3 Inhibitory effects (MIC, µg/ml) of *T. orientalis* inflorescence fractions from plants collected from different regions in Thailand against bacterial strains. **Note:** *Each sample was tested in duplicate: S1 = Sample 1 and S2 = Sample 2 **Abbreviation of bacterial strains: SA ATCC 43300 = *Staphylococcus aureus* ATCC 43300 (methicillin-resistant), SA ATCC 25923 = *Staphylococcus aureus* ATCC 25923, PA ATCC 27853 = *Pseudomonas aeruginosa* ATCC 27853 (AmpC *β*-lactamase producing strain) and AB ATCC 19606 = *Acinetobacter baumannii* ATCC 19606

## Additional Information and Declarations

### Data Availability

The following information was supplied regarding data availability: The raw measurements are available in the Supplementary Tables.
