# Supplementary material for: Cannabinoids from inflorescences fractions of Trema orientalis (L.) Blume (Cannabaceae) against human pathogenic bacteria
Source: PeerJ. 2021 May 13;9:e11446. doi: 10.7717/peerj.11446 (PMC8126263; doi:10.7717/peerj.11446)
Supplement: Supplemental Information 1 — Note: *Each sample was tested in duplicate: S1 = Sample 1 and S2 = Sample 2 **Abbreviation of bacterial strains: SA ATCC 43300 = Staphylococcus aureus ATCC 43300 (methicillin-resistant), SA ATCC 25923 = Staphylococcus aureus ATCC 25923, PA ATCC 27853 = Pseudomonas aeruginosa ATCC 27853 (AmpC β-lactamase producing strain) and AB ATCC 19606 = Acinetobacter baumannii ATCC 19606. [file peerj-09-11446-s001.docx]

**Supplemental Table 1:**

Inhibition zone (mm) of *T. orientalis* inflorescence fractions from plants collected from different regions in Thailand against bacterial strains.

| **Thai Floristic regions*** | **Inhibition zone (mm)**** | | | | | | | | | | |
| --- | --- | --- | --- | --- | --- | --- | --- | --- | --- | --- | --- |
|  | **SA ATCC 43300** | |  | **SA ATCC 25923** | |  | **PA ATCC 27853** | |  | **AB ATCC 19606** | |
|  | **S1** | **S2** |  | **S1** | **S2** |  | **S1** | **S2** |  | **S1** | **S2** |
| Northern | 9.5 | 8 |  | 10 | 11 |  | 12.5 | 12.8 |  | 9.5 | 8 |
| Southeastern or Eastern | 9 | 8.8 |  | 9 | 9 |  | 12.2 | 12.5 |  | 9 | 8 |
| Southern or Peninsular | 9.8 | 10.2 |  | 10 | 11.6 |  | 13.8 | 14 |  | 9 | 8.8 |

**Note:**  *Each sample was tested in duplicate: S1=Sample 1 and S2=Sample 2

**Abbreviation of bacterial strains: SA ATCC 43300 = *Staphylococcus aureus* ATCC 43300 (methicillin-resistant), SA ATCC 25923 = *Staphylococcus aureus* ATCC 25923, PA ATCC 27853 = *Pseudomonas aeruginosa* ATCC 27853 (AmpC β-lactamase producing strain) and AB ATCC 19606 = *Acinetobacter baumannii* ATCC 19606
