# Supplementary material for: Cannabinoids from inflorescences fractions of Trema orientalis (L.) Blume (Cannabaceae) against human pathogenic bacteria
Source: PeerJ. 2021 May 13;9:e11446. doi: 10.7717/peerj.11446 (PMC8126263; doi:10.7717/peerj.11446)
Supplement: Supplemental Information 2 — SA ATCC 25923: Staphylococcus aureus ATCC 43300. SA ATCC 43300: Staphylococcus aureus ATCC 43300. KP * ATCC 700603: K. pneumoniae ATCC 700603 and PA * ATCC 27853: Pseudomonas aeruginosa ATCC 27853. These strains were used as control species; the inhibition zone in each antibiotic was within the quality control ranges set by the CLSI (2019)*. NT abbreviation was refer to not tested. *The quality control ranges set by the CLSI (2019): For K. pneumoniae strain ATCC 700603, the different clear zone diameter of antibiotics including ceftazidime (10–18 mm), cefotaxime (17–25 mm), and ceftriaxone (16–24 mm). For P. aeruginosa strain ATCC 27853, the different clear zone diameter of antibiotics including piperacillin/tazobactam (25–33 mm), amikacin (18–26 mm), and ciprofloxacin (25–33 mm). [file peerj-09-11446-s002.docx]

**Supplemental Table 3:**

The inhibition effects of standard antibiotics with control species by the CLSI (2019).

| **Standard antibiotics** | **SA**  **ATCC 25923** | **SA**  **ATCC 43300** | **KP***  **ATCC 700603** | **PA***  **ATCC 27853** |
| --- | --- | --- | --- | --- |
| Piperacillin/tazobactam (100/10 µg) | 28 | 25 | NT | 28 |
| Amikacin (30 µg) | 23 | 30 | 20 | 23 |
| Ceftriaxone (30 µg) | NT | NT | 18 | NT |
| Cefotaxime (30 µg) | NT | 25 | 17 | NT |
| Ceftazidime (30 x) | 26 | NT | 13 | 26 |
| Ciprofloxacin (5 µg) | 30 | 30 | NT | 30 |
| Imipenem (10 µg) | 22 | NT | 29 | 22 |
| Trimethoprim/sulfamethoxazole (1.25/23.75µg) | NT | 30 | NT | NT |
| Vancomycin (30 µg) | NT | 18 | NT | NT |

**SA ATCC 25923**: *Staphylococcus aureus* ATCC 43300. **SA ATCC 43300**: *Staphylococcus aureus* ATCC 43300. **KP^*^ ATCC 700603**: *K. pneumoniae* ATCC 700603 and **PA^*^ ATCC 27853**: *Pseudomonas aeruginosa* ATCC 27853. These strains were used as control species; the inhibition zone in each antibiotic was within the quality control ranges set by the CLSI (2019)*. NT abbreviation was refer to not tested.

*The quality control ranges set by the CLSI (2019): For *K. pneumoniae* strain ATCC 700603, the different clear zone diameter of antibiotics including ceftazidime (10-18 mm), cefotaxime (17-25 mm), and ceftriaxone (16-24 mm). For *P. aeruginosa* strain ATCC 27853, the different clear zone diameter of antibiotics including piperacillin/tazobactam (25-33 mm), amikacin (18-26 mm), and ciprofloxacin (25-33 mm).
